# Azole Resistance in *Aspergillus fumigatus*: A Consequence of Antifungal Use in Agriculture?

**DOI:** 10.3389/fmicb.2017.01024

**Published:** 2017-06-07

**Authors:** Sarah Berger, Yassine El Chazli, Ambrin F. Babu, Alix T. Coste

**Affiliations:** Institute of Microbiology, University Hospital Center, University of LausanneLausanne, Switzerland

**Keywords:** agriculture, antifungals, azole, *Aspergillus fumigatus*, aspergillosis, resistance, CYP51A, TR34/L98H

## Abstract

Agricultural industry uses pesticides to optimize food production for the growing human population. A major issue for crops is fungal phytopathogens, which are treated mainly with azole fungicides. Azoles are also the main medical treatment in the management of *Aspergillus* diseases caused by ubiquitous fungi, such as *Aspergillus fumigatus*. However, epidemiological research demonstrated an increasing prevalence of azole-resistant strains in *A. fumigatus*. The main resistance mechanism is a combination of alterations in the gene *cyp51A* (TR34/L98H). Surprisingly, this mutation is not only found in patients receiving long-term azole therapy for chronic aspergillosis but also in azole naïve patients. This suggests an environmental route of resistance through the exposure of azole fungicides in agriculture. In this review, we report data from several studies that strongly suggest that agricultural azoles are responsible for medical treatment failure in azole-naïve patients in clinical settings.

## Introduction

Agriculture undergoes many challenges comprising pests, diseases and abiotic stresses, which drastically decrease crop yield (Ricroch et al., 2016). This contributes to important losses to farmers and threatens global food production capacity. A wide range of viral, fungal and bacterial plant pathogens has been known to contribute for these losses. As an example, Cassava Mosaic and Brown Streak virus diseases affect food crops in Africa (Food and Agriculture Organization, 2016) while plant fungal pathogens are responsible for numerous diseases including septoria leaf blotch, powdery mildews and rusts (Price et al., 2015).

For the last centuries, chemical industries have provided solutions to deal with plant infections and lower crop losses. The development of antiviral, antibiotics and antifungals has allowed overcoming the effect of phytopathogens but may have considerable negative impact on the environment, mainly with regard to the contamination of surface water and groundwater (Azevedo et al., 2015). Focusing on fungal pathogens, there are a number of antimycotic compounds available to control the spread of these pathogens. However, it is azole antifungals that are the preferred treatment owing to their effectiveness against a broad range of fungi (Price et al., 2015). In addition to controlling the spread of agricultural fungal pathogens, antifungal agents of the azole class play an important role in the management of human fungal diseases (Snelders et al., 2012).

Among fungal pathogens, many are both present in the environment and in clinic. In this review, we will focus on *Aspergilli*, especially, *Aspergillus fumigatus*, a saprophytic fungus, developing on decaying vegetation, in soil, and producing volatile conidia (spores) carried in the air. Such spores are constantly inhaled by humans and in certain conditions could be the cause of one of the most frequent human fungal diseases, aspergillosis which includes allergic, chronic and invasive aspergillosis (Klich, 2007). In clinic, long-term azole therapy of aspergillosis led to the emergence of azole resistance in *A. fumigatus* over the last decades (Hagiwara et al., 2016). Surprisingly, azole resistance has also emerged in azole-naive patients. This observation may be explained by a second route of resistance development through environmental exposure of *A. fumigatus* to azole fungicides used in the field. This leads to our main question: *<<Does the massive use of azoles in agricultural practice lead to antifungal resistance among human pathogens, impairing medical treatment?>>*.

To address this problem, this review was organized according to the following topics: In the first part, we will discuss the use of different azoles in agricultural settings and their mode of action against plant fungal pathogens. Secondly, we will focus on the clinical use of azoles to treat patients affected with *A. fumigatus*, and finally we will present some studies in favor of a link between azole resistance due to antifungal use in agriculture and its side effects on human health.

## Use of Azole Fungicides in Agriculture

Fungal infections were fought with natural fungicides until the middle of the 20th century when synthetic fungicides were introduced. The treatments used from the 17th century, were mainly mixtures of natural elements or compounds used to overcome blights, bunts or mildews. Arsenic, brine and copper sulfate were treatments for different cereal seeds. Copper sulfate is still used as the main component of Bordeaux mixture, which is a preventive treatment against mildews in vines, tomatoes, and potatoes (Morton and Staub, 2008).

In the 1940s, chemically synthesized compounds, like dithiocarbamates, were released onto the market. They presented a higher efficiency than earlier compounds, which required up to 20 kg/ha to be efficient. Effectively, the dithiocarbamates along with phtalimides, fentins and benzimidazoles were used in quantities ranging from 1.5 to up to 3 kg per hectare (Morton and Staub, 2008). However, benzimidazoles were extensively used and resistance was reported in multiple fungi after only 3–4 seasons of spraying (Russell, 1995).

Azole, a second generation of chemical antifungals was introduced in the 1970s. Azoles are unsaturated aromatic molecules containing at least one nitrogen atom. Today, they are the most used antifungals because of their high efficiency and broad spectrum activity (Price et al., 2015) (**Table 1**). The current use rates for many triazoles, which are the largest class of azole antifungals used nowadays, are below/or around 100 g/ha of plant surface (Morton and Staub, 2008; Azevedo et al., 2015).

**Table 1:** Different azole compounds (mainly triazoles) released on the market in the early 1970s to 2000s.

Year	Common name of compounds	Treatment^†^
1973	Triadimefon	Broad
	Imazalil (imidazole)	Post-harvest and seed
1977	Triadimenol	Seed treatment
	Prochloraz (imidazole)	Cereal fungicide
1979	Propiconazole, Bitertanol	Broad
1982	Triflumizole	Broad
1983	Flutriafol, Diniconazole,	Broad
	Flusilazole	
1986	Hexaconazole,	Broad
	Cyproconazole,	Broad/foliar and seed
	Tebuconazole^∗^	
1988	Difenoconazole,	Broad/foliar and seed
	Tetraconazole	
1990	Epoxiconazole^∗^	Broad/cereals
1992	Metconazole,	Broad
	Fluquinconazole,	Broad, foliar and seed
	Triticonazole	
2002	Prothioconazole^∗^	Broad


*^†^The third column shows the use of the different antifungals (Morton and Staub, 2008). ^∗^Prothioconazole, epoxiconazole and tebuconazole are the most used antifungals in the United Kingdom, Netherlands, and Denmark (Kleinhauf et al., 2013).*

Azole antifungals are part of the Sterol Biosynthesis Inhibitors (SBI) (Azevedo et al., 2015). They affect the same target in fungal cells. The lanosterol 14α-demethylase (also known as ERG11) or CYP51A in *A. fumigatus* is a P450 enzyme family member. It is an important enzyme, which regulates ergosterol biosynthetic pathway. Ergosterol is an essential component that ensures the permeability and fluidity of the cell membrane (Price et al., 2015). The drug binds to the enzyme, forming a catalytically inactive complex. Loss of activity of CYP51A causes interruption of ergosterol synthesis and a toxic high rate of demethylated lanosterol in the cell (Price et al., 2015). The effect of the drug results in a disrupted cell transport and membrane structure. Fungicides, unlike their name might suggest may only be fungistatic, as they do not kill fungal cells (Price et al., 2015). For *Aspergilli*, their fungicidal effect was shown to be species and strain dependent. Resistance issues reported in agriculture have been addressed either by increasing the doses of fungicides sprayed, or by mixing different fungicide types (Russell, 1995). Emergence of resistance is not systematically obtained after antifungal drug exposure as shown by the work of Kano et al. (2015) who sprayed fields twice a year with tetraconazole. Lack of antifungal resistance may be due to the low frequency of azole field treatment or the use of only one type of azole drug (tetraconazole) as suggested also by the authors in contrast to real agricultural practice.

In conclusion, the broad treatment of crops with azoles leads to an exposure of all fungi present in the field. *Aspergilli* are therefore exposed to the fungicide in the environment, leading to resistance issues that might have consequences on human health (Snelders et al., 2009).

## Use of Azoles in Clinic and Resistance Development Through Patient Exposure

In clinic, the increasing number of fungal infections causes a high rate of morbidity and mortality in immunocompromised patients (Hagiwara et al., 2016). This includes patients with hematological malignancy, pulmonary diseases, solid-organ or hematopoietic stem cell transplantation, and patients treated with corticosteroids (Verweij et al., 2009). Common fungal infections like chronic or acute invasive aspergillosis, the allergic bronchopulmonary aspergillosis, and aspergilloma are caused by *Aspergillus* (Snelders et al., 2012). Patients develop these infections by inhaling airborne *Aspergillus* spp. conidia, which are normally eliminated by the immune system but can cause invasive diseases in immunocompromised individuals. Approximately 30 species of *Aspergillus* spp. are involved in *Aspergillus* spp. infections, but the most frequent species is *Aspergillus fumigatus* (*A. fumigatus*) (Lelièvre et al., 2013).

To prevent and treat *Aspergillar* infections, four classes of antifungal drugs are used: pyrimidine, echinocandin, polyene, and azoles (Hagiwara et al., 2016). Azoles are major agents in the treatment and prophylaxis of aspergillosis (Lelièvre et al., 2013; Hagiwara et al., 2016). Itraconazole, voriconazole and posaconazole are the most widely used azole antifungal drugs. Voriconazole is prescribed for the primary treatment of aspergillosis while posaconazole and itraconazole cause a reduction of invasive fungal infections in neutropenic patients with acute myeloid leukemia and myelodysplastic syndrome (Verweij et al., 2009).

The increasing use of azoles in the management of *Aspergillus* diseases led to resistances. Resistance is confined to the individual and no transmission between patients has been reported. Azole-resistant *A. fumigatus* strains were first reported in the late 1990s in patients from the United States who had received long-term itraconazole therapy (Lelièvre et al., 2013). Since then, many cases of resistant strains were discovered in Europe and elsewhere, especially in the Netherlands and in the UK where the prevalence reached 6 and 5%, respectively (Lelièvre et al., 2013). These resistances are especially found in patients who received azole drug for a long period to treat chronic aspergillosis. Azole-resistant isolates are now identified in Norway, Spain, Belgium, Denmark, France, Germany, India, Iran, Portugal, Brazil, the Czech Republic, Turkey, Japan, Kuwait, Taiwan, Australia and China (Rivero-Menedez et al., 2016; Garcia-Rubio et al., 2017).

### Mutation in the *cyp51A* Gene

**FIGURE 1:**
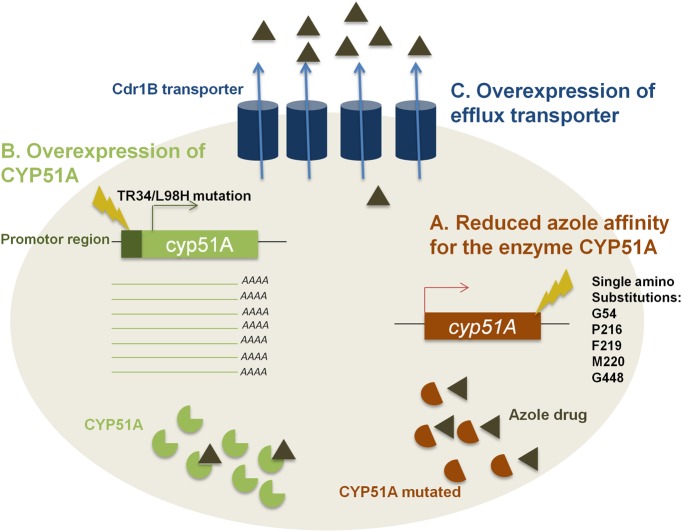
The three mechanisms of resistance to azole drugs. **(A)** Presence of mutations responsible for substitution causing structural modifications of CYP51A enzyme leading to a reduced azole affinity. **(B)** Overexpression of *cyp51A* gene due to a 34-bp insertion in its promoter region combined with a substitution at codon 98 of leucine to histidine (TR34/L98H) causing an increase in cellular CYP51A level. **(C)** Overexpression of the genes coding for efflux transporter causing a reduced intracellular accumulation of azole drug.

The fungus *A. fumigatus* and the other agricultural fungal pathogens share the same mechanisms of azole resistance (Price et al., 2015). This includes several mutations in the *cyp51A* gene. These mutations are responsible for substitutions causing a structural modification of the CYP51A enzyme. This leads to an alteration in azole’s affinity for the enzyme causing azole tolerance (**Figure 1**). Single amino acid substitutions like G54, P216, F219, M220, and G448 in the CYP51A protein lead to azole tolerance as shown in **Figure 1A** (Hagiwara et al., 2016). These resistance mutations appear in patients with aspergilloma or other *Aspergillus* cavities that received long-term azole therapy.

### Overexpression of *cyp51A*

In addition to these point mutations, changes in the promoter region of the *cyp51A* gene by the insertion of tandem repeats or transposable elements lead to an overexpression of CYP51A (**Figure 1B**). Indeed, the 34 base-pair tandem repeat (TR34) which is always found together with a lysine to histidine substitution at codon 98 (TR34/L98H) induces an up to eightfold increase of the normal expression of CYP51A (Lelièvre et al., 2013). It has been hypothesized that the increase of mRNA levels correlates with the augmentation in cellular CYP51A levels causing a reduction in azole sensitivity (Price et al., 2015).

### Efflux Transporters

Even though CYP51A is the main target of azole compounds, other enzymes are capable of causing resistance. Efflux pumps, including ATP-binding cassette (ABC) transporters and major facilitator superfamily (MFS) transporters are necessary in eukaryotic organisms to remove toxins out of the cell (Jasinski et al., 2003). Therefore, overexpression of these genes leads to resistance against azole as the intracellular concentration of the fungicide decreases (**Figure 1C**). The fungus *A. fumigatus* contains at least 49 genes encoding the ABC transporters. Among them, it has been shown that 12 genes present a high homology with *S. cerevisiae* PDR5 and PDR15 proteins that are involved in azole resistance too (Hagiwara et al., 2016). The Cdr1B efflux transporter, a member of PDR subfamily, was shown to be overexpressed in azole-resistant isolates. The deletion of *cdr1B* gene in a resistant strain results in increased sensitivity to itraconazole (Hagiwara et al., 2016). This means that Cdr1B is primordial for azole resistance in *A. fumigatus* (Hagiwara et al., 2016). Recently, it has been demonstrated that deletion mutants of two other distinct ABC transporters (AtrF, AtrI) and a major facilitator superfamily transporter (MdrA) also showed sensitivity to azole drugs (Meneau et al., 2016).

## Link Between Azole Resistance in Agriculture and Clinic

Surprisingly, resistance to azoles is confined not only to patients undergoing azole therapy, but also in many azole naïve patients, with no known prior exposure to azole drugs (Snelders et al., 2009). As inhalation of airborne *Aspergillus* spores is the common route of infection for *Aspergillus* diseases, it has been hypothesized that the resistance could have been acquired from a common environmental source. For example, if azole-resistant *A. fumigatus* is present in our environment, patients could inhale resistant spores and subsequently develop azole-resistant diseases (Snelders et al., 2009).

Azoles are found to be actively persistent for several months in many ecological niches such as agricultural soil and aquatic environments. As reviewed above, azoles are commonly used in agriculture as fungicides and also for the preservation of a variety of materials such as wood. Hence, it was hypothesized that the fungal exposure to azole compounds in the environment results in a cross-resistance to medical triazoles (Chowdhary et al., 2013).

To validate this hypothesis, two studies sampled azole resistant *A. fumigatus* strains from patients with previous exposure to azoles and from the environment (flowerbeds, compost, leaves, plant seeds, soil samples of tea, paddy fields, hospital surroundings, and aerial samples of hospitals) (Chowdhary et al., 2013; Verweij et al., 2016) and studied the different mechanisms of resistance. Surprisingly, a combination of alterations previously described in patients, were found not only in clinical samples but also in the environmental isolates. This includes essentially mutations in the *cyp51A* gene (TR34/L98H, G448S and TR46/Y121F/T289A). In addition, a very recent study, clearly highlighted that an acquisition of mutation in the azole target by the environmental strains leads to cross resistance between the azole antifungals in the environment and the clinic (Ren et al., 2017). Among 144 soil samples treated with many azoles (epoxiconazole, tebuconazole, propiconazole, hexaconazole, metconazole), Ren et al. found that 5.8% of the analyzed samples were resistant to medical azoles (Ren et al., 2017). Two samples were resistant to voriconazole with most frequent mutations found in the environmental samples (TR46/Y121F/T289A). One sample was resistant to itraconazole with mutation TR34/L98H/S297T/F495I.

TR34/L98H was the most common mutation in both environmental and clinical samples (Rivero-Menedez et al., 2016; Verweij et al., 2016). All *A. fumigatus* isolates with this mutation showed cross-resistance to medical and agricultural triazoles. Docking studies revealed that both medical and agricultural triazoles have similar molecular structures and adopt similar conformations (Chowdhary et al., 2013). TR34/L98H mechanism was found to be widespread in The Netherlands and is also found in other European countries, the Middle East, Asia, Africa, Australia and, most recently, North and South America. A wide range of *cyp51A* mutations were reported in both clinical and environmental isolates in Austria, Belgium, Denmark, France, Germany, Greece, Italy, The Netherlands, Poland, Portugal, Romania, Spain, Sweden, Turkey and the United Kingdom (Rivero-Menedez et al., 2016). Recently, azole resistance has also been found in Norway, Iran, Brazil, the Czech Republic, Japan, Kuwait and Taiwan (Garcia-Rubio et al., 2017).

The following studies were done to gain insights into the origin and spread of this resistance. Genotyping of epidemiologically and geographically unrelated strains showed a lower genetic diversity among isolates harboring TR34/L98H and TR46/Y121F/T289A compared to wild-type isolates, which suggests that each mutation might have originated from a common ancestor genotype (Verweij et al., 2016). The azole-resistant isolates from clinical and environmental samples exhibited identical phenotype and mechanism of resistance. Also, microsatellite typing of the isolates showed that the environmental and clinical azole resistant isolates clustered together, indicating genetic relatedness, also suggesting the possibility for a common ancestor (Snelders et al., 2009).

Even if novel point mutations like G54 and M220 were found exclusively in clinical samples (Rivero-Menedez et al., 2016) several studies provide evidence that (i) mutations acquired in the fields confere resistance against agricultural azole but also against clinical azoles, and (ii) patients with invasive aspergillosis due to azole-resistant *A. fumigatus* might acquire the fungus from the environment.

## Conclusion and Outlook

**FIGURE 2:**
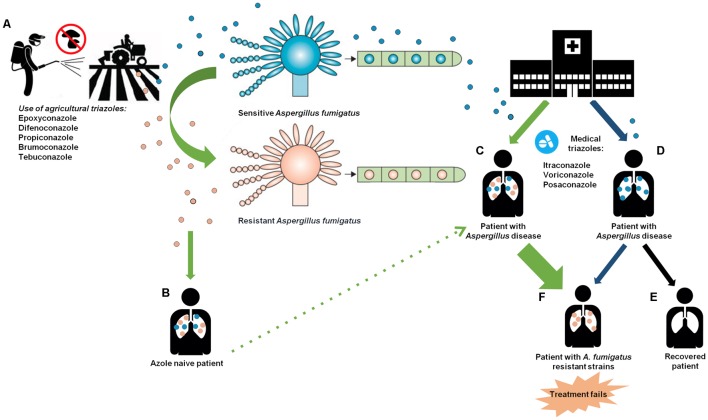
The environmental and clinical routes of azole resistance development. **(A)** The exposure of *A. fumigatus* to azole compounds in agriculture may create mutations in conidia inducing a resistant phenotype. **(B)** Azole naive patient becomes ill upon inhalation of airborne resistant and sensitive *A. fumigatus* strains. **(C)** The azole naive patient is treated with medical triazoles creating a persistent pressure of azole leading to the selection of resistant strains. **(D)** Patients infected by inhaling sensitive *A. fumigatus* molds receive long-term azole therapy. **(E)** A small proportion of patient who received long-term azole therapy without developing resistant *A. fumigatus* strains (black arrow). **(F)** Patients with *A. fumigatus* resistant strains that have developed through long-term azole therapy (blue arrow) or by the use of azole in agriculture (green arrow). This induces a failure in the management of *Aspergillus* diseases. Adapted from Verweij et al. (2009).

Azole resistance in *A. fumigatus* develops either during treatment in the clinic or following intensive agricultural practice as summarized in **Figure 2**. The environmental route of resistance development has been reported since 2007 and is rapidly appearing worldwide. Even though the highest rates of triazole resistance have been described in Europe, several cases have been reported in every continent, and new resistance mechanisms are being described (Rivero-Menedez et al., 2016), which is highly problematic for agriculture. Simply prohibiting the use of the fungicides is not feasible, as this might result in crop disease epidemics and subsequent food shortages and economic losses. Hence, there is a necessity to change the practice in the field. These could include prudent and restricted use of fungicides in terms of rotation of products, doses and periods of application. In those cases where resistance to treatment is observed, either the dosage can be increased or alternative fungicides can be used.

There is also a further need for surveillance, collection of precise information and extensive programs of resistance monitoring into the agricultural field to investigate the size of the emerging problem of azole resistance. Research at epidemiological level could indicate the geographic variations in the occurrence of resistance and would facilitate identification of the regions with high incidence of resistance (SCCNFP, 2003). Furthermore, research at the laboratory level should aim at understanding the conditions under which resistance mechanisms develop in the environment and which *Aspergillus* morphotype is most prone to develop mechanisms of resistance (Snelders et al., 2012).

Additionally, the emerging resistances become a threat to millions of people across the globe because the same resistant strains of *A. fumigatus* observed in the environment are also found in the clinic. Due to the fact that resistance is associated with treatment failure, it is necessary to optimize agricultural practices. In parallel, new, safe and effective classes of antifungals and compounds that inhibit the resistance mechanisms in fungi have to be developed in both agriculture and clinic. Modification, combination, and repurposing of current antifungals and other FDA-approved drugs and seeking out new sources of antifungal agents could serve as potential antifungal leads (Ngo et al., 2016).

Another issue of the extensive use of azoles in agriculture could be the emergence of resistance in non-environmental fungi. Non-ubiquitous fungi present in the human flora such as *Candida* spp., are constantly in contact with environmental azoles.

Indeed, this contact occurs inside the body by ingestion of azole-contaminated food, or by interaction with insects or even by inhaling recently sprayed azoles on crops. However, this hypothesis requires further investigations.

Resistance issues are not only confined to fungi, but also to viral and bacterial pathogens. Finding new drugs and ways to control microbial pathogens as well as adapting the current strategies is a major concern for human health.

## Author Contributions

SB, YEC, AB did a literature review exercise in the context of their master at the University of Lausanne under the supervision of AC.

## Conflict of Interest Statement

The authors declare that the research was conducted in the absence of any commercial or financial relationships that could be construed as a potential conflict of interest. The reviewer LA-F and handling Editor declared their shared affiliation, and the handling Editor states that the process nevertheless met the standards of a fair and objective review.
